# Multiplex Digital Methylation‐Specific PCR for Noninvasive Screening of Lung Cancer

**DOI:** 10.1002/advs.202206518

**Published:** 2023-04-11

**Authors:** Yang Zhao, Christine M. O'Keefe, Kuangwen Hsieh, Leslie Cope, Sonali C. Joyce, Thomas R. Pisanic, James G. Herman, Tza‐Huei Wang

**Affiliations:** ^1^ Department of Biomedical Engineering Johns Hopkins University Baltimore MD 21287 USA; ^2^ Department of Mechanical Engineering Johns Hopkins University Baltimore MD 21218 USA; ^3^ Department of Oncology Johns Hopkins University Baltimore MD 21287 USA; ^4^ The UPMC Hillman Cancer Center University of Pittsburgh Pittsburgh PA 15232 USA; ^5^ Division of Hematology and Oncology Department of Medicine, University of Pittsburgh Medical Center Pittsburgh PA United States; ^6^ Johns Hopkins Institute for NanoBioTechnology Johns Hopkins University Baltimore MD 21218 USA

**Keywords:** cancer diagnostics, digital PCR, DNA methylation, epigenetics, liquid biopsy, lung cancer, microfluidics

## Abstract

There remains tremendous interest in developing liquid biopsy assays for detection of cancer‐specific alterations, such as mutations and DNA methylation, in cell‐free DNA (cfDNA) obtained through noninvasive blood draws. However, liquid biopsy analysis is often challenging due to exceedingly low fractions of circulating tumor DNA (ctDNA), necessitating the use of extended tumor biomarker panels. While multiplexed PCR strategies provide advantages such as higher throughput, their implementation is often hindered by challenges such as primer‐dimers and PCR competition. Alternatively, digital PCR (dPCR) approaches generally offer superior performance, but with constrained multiplexing capability. This paper describes development and validation of the first multiplex digital methylation‐specific PCR (mdMSP) platform for simultaneous analysis of four methylation biomarkers for liquid‐biopsy‐based detection of non‐small cell lung cancer (NSCLC). mdMSP employs a microfluidic device containing four independent, but identical modules, housing a total of 40 160 nanowells. Analytical validation of the mdMSP platform demonstrates multiplex detection at analytical specificities as low as 0.0005%. The clinical utility of mdMSP is also demonstrated in a cohort of 72 clinical samples of low‐volume liquid biopsy specimens from patients with computed tomography (CT)‐scan indeterminant pulmonary nodules, exhibiting superior clinical performance when compared to traditional MSP assays for noninvasive detection of early‐stage NSCLC.

## Graphical abstract

This paper describes the development and analytical/clinical validation of the first mdMSP platform for the simultaneous analysis of four DNA methylation biomarkers for liquid‐biopsy‐based detection of early‐stage NSCLC. The demonstrated analytical sensitivity/specificity and clinical utility indicate the potential of mdMSP platform to be a complementary method for improved CT screening of lung cancer.

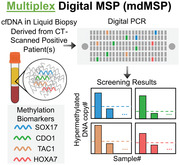

## 1 Introduction

Liquid biopsies have gained considerable attention during the past decade as an attractive means of achieving noninvasive detection, screening, and monitoring of disease, and cancer in particular. Cancer diagnostics based on liquid biopsies is generally achieved through detection of cancer‐specific genetic or epigenetic aberrations in cfDNA that is released from tissues throughout the entire body.^[^ ^1^ ^]^ Blood, together with body fluids like urine or stool, offer numerous advantages over traditional biopsies such as comprehensive tissue sampling and capture of intratumor heterogeneity, repeatable sample collection throughout the course of disease and access in vulnerable individuals.^[^ ^2^ ^]^ Aberrant DNA methylation is a notable early event in carcinogenesis that continues to be widely investigated as a means of early detection of many types of cancer.^[^ ^1^, ^3^ ^]^ Though promising, tumor‐derived DNA with such molecular alterations are rare in liquid biopsy specimens and typically exists among a high background of DNA from healthy tissues, often resulting in variant allele frequencies as low as 0.01%.^[^ ^4^ ^]^ Furthermore, studies have shown that there may be as few as 1–7 copies of tumor‐derived DNA in each mL of plasma.^[^ ^5^ ^]^ Therefore, detection of these rare and infrequent tumor‐specific DNA methylation biomarkers necessitates the use of assays that offer high sensitivity, specificity and detection efficiency, preferably at sufficiently low‐cost for potential use in routine diagnostic applications.

Current methylation analysis techniques for liquid biopsy‐based diagnostics generally rely on one of two detection methods: PCR‐based techniques or next‐generation sequencing (NGS). PCR‐based methods, such as droplet digital methylation‐specific PCR (ddMSP), can achieve single‐copy sensitivity and are suitable for detecting low copy numbers of tumor DNA from cancer patients by compartmentalizing samples into droplets that contain no more than a single target molecule or locus. However, most digital approaches are limited to detection of 1–2 targets due to limitations in the number of fluorescence channels in most commercial instruments, such as QuantStudio (Thermo Fisher Scientific) and QX200 (Bio‐Rad).^[^ ^6^ ^]^ While early‐stage cancer detection would benefit from the increased sensitivity and specificity afforded by digital approaches, most diagnostic assays for methylation detection are developed using standard bulk PCR methods, such as methylation‐specific PCR (MSP) and MethyLight.^[^ ^7^ ^]^ Examination of each biomarker by standard singleplex bulk PCR significantly increases labor costs, time and sample volume. On the other hand, the development of multiplex assays is also often complicated by challenges associated with complex PCR mixtures, such as primer‐dimers and PCR competition. Furthermore, these issues can be particularly problematic in the development of methylation detection assays, which typically target high GC content regions that can exacerbate primer dimer or secondary structures, leading to compromised performance.^[^ ^8^ ^]^ Alternatively, NGS‐based detection provides analysis of many loci in parallel, but often struggles to detect circulating tumor DNA (ctDNA) from early‐stage tumors with limited ctDNA mutant allele fractions of 0.1% or lower.^[^ ^9^ ^]^ Although several well‐documented approaches have recently improved diagnostic sensitivity through the use of a multianalyte paradigm (e.g., CancerSEEK) or extremely large biomarker panels (e.g., cfMedDip‐seq and Galleri), these techniques remain expensive, highly prone to overfitting and likely impractical for routine diagnostic applications.^[^ ^10^ ^]^ There thus remains a need for approaches that can evaluate multiple, validated biomarkers with high sensitivity in a cost‐efficient manner.

Lung cancer is one of the most common and lethal cancers worldwide.^[^ ^11^ ^]^ NSCLC accounts for 85% of all lung cancer cases and has a 5 year survival rate of only 25%.^[^ ^12^ ^]^ While low‐dose CT screening has been reported to provide a roughly 20% reduction in lung cancer mortality, it suffers from a high false discovery rate of more than 96%, which can lead to complications and even death associated with follow‐up procedures.^[^ ^13^ ^]^ There thus remains a critical need for techniques to improve the accuracy of NSCLC screening. Numerous studies, by us and others, have reported the potential use of DNA methylation biomarkers for detection of early‐stage NSCLC.^[^ ^14^ ^]^ We previously demonstrated that [singleplex] qPCR‐based real‐time MSP assays could be used to reliably detect a panel of six DNA methylation biomarkers in plasma derived from liquid biopsies of NSCLC cancer patients, with potential use for early‐stage NSCLC detection.^[^ ^15^ ^]^ The results of these studies indicated that four biomarkers in particular (*SOX17*, *CDO1*, *TAC1*, and *HOXA7*) provided the best performance in terms of overall clinical sensitivity and specificity.

In this study, we aimed to develop the first mdMSP assay to analyze this panel of four methylation biomarkers as a means of improving detection of NSCLC in liquid biopsy specimens. The mdMSP platform employs a microfluidic device comprising four independent but identical digitization modules that collectively house a total of 4 × 10 040 = 40 160 nanowells and allow for multiplexed detection using four differentially‐colored fluorescence probes corresponding to each of four targets in the panel. This microfluidic array can digitize four samples per run and analyze thousands of individual molecules while limiting reagent consumption and providing absolute quantification of amplified template molecules. Furthermore, the device does not require additional customized heaters or imaging systems and is compatible with off‐the‐shelf commercial instrumentation. In this work, we describe fabrication of the mdMSP platform for the implementation of multiplex liquid biopsy assays. The analytical performance of the mdMSP assay is then validated and compared with corresponding bulk assays in both singleplex and multiplex formats. Last, we demonstrate the potential clinical utility of the mdMSP platform in a cohort of 72 liquid biopsy specimens from cancer‐positive and healthy controls with CT‐positive indeterminant pulmonary nodules, achieving superior clinical performance when compared to the traditional bulk MSP formats. Overall, we show that mdMSP can potentially serve as a useful paradigm demonstrating that the development of multiplexed assays can be surprisingly facilitated by employing a digital format.

## 2 Results

### 2.1 Overview of the Multiplex Digital Methylation‐Specific PCR Assay

In our previous work, we analyzed tissue sample data from the Cancer Genome Atlas project (TCGA) to identify 64 genes that showed lung cancer‐specific hypermethylation within the CpG‐islands of their promoters.^[^ ^16^ ^]^ We subsequently developed singleplex, real‐time methylation‐specific PCR [MethyLight]^[^ ^7c^ ^]^ assays targeting the promoters of four of these genes (*SOX17*, *TAC1*, *CDO1*, and *HOXA7)*, the results of which could be combined to distinguish between CT‐scan positive patients with NSCLC from those with no or benign disease.^[^ ^15b^ ^]^ The overall goal of this study was to design and validate an improved platform that leverages sensitive digital multiplex analysis of these four DNA methylation biomarkers and assess its clinical performance in plasma derived from liquid biopsies of high‐risk patients with indeterminant lung nodules identified by low‐dose CT screening. To achieve this goal, we developed a microfluidic platform capable of rapidly discretizing template DNA into thousands of nanochambers. Beyond improving quantitation, analytic sensitivity and specificity, as well as sample throughput, we additionally reasoned that the digitization of template molecules could ease the development of the multiplex assay itself by minimizing PCR competition between the targets. To enable simultaneous multiplex methylation detection, we selected four fluorescent dyes (FAM, HEX, Texas Red and Cy5) with minimal spectral overlap to label each of four different probes, thereby allowing simultaneous identification of the four targets within a single digital array.

**Figure 1:**
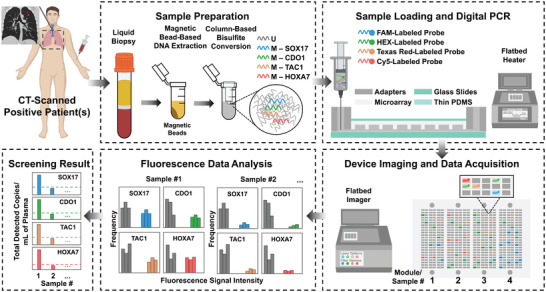
Overview of mdMSP for noninvasive screening for early‐stage lung cancer. DNA is extracted from liquid biopsy specimens derived from CT‐scan positive patients. Following bisulfite conversion, the DNA sample is loaded into one module of a microfluidic chip. Digital PCR is performed on a flatbed heater in the presence of target‐specific, fluorescently‐labeled TaqMan probes to achieve simultaneous detection of four DNA targets. The microfluidic device is imaged with a standard flatbed imager, which is equipped with combinations of lasers and filters for the four fluorophores, FAM, HEX, Texas Red and Cy5. Last, the resulting images are analyzed using our customized software program to evaluate the intensity of the fluorescence generated from the four TaqMan fluorophores. The total number of detected copies of each biomarker per mL of plasma is summarized into a histogram to determine appropriate thresholds for optimizing assay performance. The dashed lines represent individual thresholds, which were selected based on desired sensitivity and specificity. Some elements in this figure were created with BioRender.com.

A general overview of the mdMSP assay is shown in **Figure** **1**. Cell‐free DNA is first extracted from 1 mL of plasma derived from peripheral blood liquid biopsy specimens using silica‐coated magnetic beads. The extracted cfDNA is then purified and undergoes bisulfite conversion (BST) according to our previously‐published protocol.^[^ ^17^ ^]^ The bisulfite conversion process is used to convert unmethylated cytosine residues to uracil while leaving methylated cytosine intact, thus directly translating DNA methylation alterations to changes in the primary sequence of the template molecules. Samples containing bisulfite‐treated cfDNA (BST‐cfDNA), corresponding to 100 µL of plasma, are then mixed with PCR reagents including multiplex primers before loading onto the device. Target identification is achieved through the use of fluorescent TaqMan probes conjugated to a different fluorophore for each respective target. Finally, PCR is then performed on chip and the device is imaged on a flatbed fluorescence scanner equipped with filters matching the emission/excitation spectra of each respective fluorophore. Positive wells are identified through fluorescence indicating amplified individual methylated epialleles, which are then enumerated by custom automated analysis software.

### 2.2 Microfluidic Device Design and Operation

**Figure 2:**
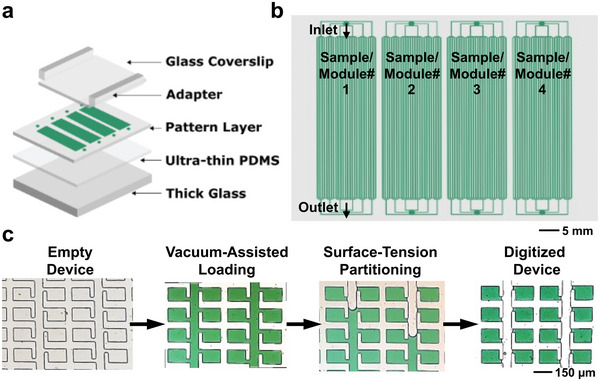
mdMSP device design and operation. a) The mdMSP device is composed of four primary layers: a PDMS‐coated glass slide, an ultra‐thin PDMS layer, a patterned PDMS layer and a thin glass coverslip between adapters accommodating inlets and outlets. b) Each mdMSP device comprises four independent modules, and each module holds 10 040 1 nL nanowells. c) Samples are rapidly drawn into the device, which has been desiccated to create a negative pressure differential. A partitioning liquid is then injected into the inlet and maintained under pressure to lock the digitized sample in place. A detailed description of the fabrication process can be found in previous publications.^[^ ^18^ ^]^

The mdMSP device utilizes a polydimethylsiloxane (PDMS) array fabricated according to a previously‐reported ultrathin soft lithography method, which mitigates sample loss during thermocycling and reduces thermal deviation.^[^ ^18^ ^]^ The device features rapid, vacuum‐assisted loading that enables sample loading and digitization to be performed in less than 15 s (**Figure** **2**b). Digitization is achieved through surface‐tension‐based partitioning by pressure‐loading an oil‐based solution through the channels. During PCR, partitioning oil remains pressurized to prevent sample from leaking back into the channels. PDMS was also added to the partitioning oil, which solidifies during PCR to prevent cross‐contamination between reaction chambers and allows the device to be readily handled without risk of contamination. In order to increase throughput, we designed a microfluidic device amenable for use with standard fluorescence instrumentation and each chip includes four independent but identical modules, housing 10 040 nanowells each, thereby allowing up to four samples to be digitized and analyzed in parallel (Figure 2a). The nanowells were designed to hold 1 nL of volume, which is small enough to allow absolute digitization of cfDNA template molecules, while retaining physical dimensions compatible with the spatial resolutions of most commercial imaging systems. Imaging was achieved using a flatbed fluorescence scanner, which uses lasers paired with emission filters matched to the respective excitation and emission spectra of each respective fluorophore. The resulting raw imaging data were then processed using a “known‐mask” mapping technique, as described previously.^[^ ^18a^ ^]^ This customized software program relies on mapping a predefined mask to the array by using four geometric user inputs and then captures the fluorescence intensity value of each well. The entire analysis procedure can be completed in less than 3 min.

### 2.3 Multiplex Digital MSP Outperforms Bulk Multiplex MethyLight

We first validated the analytical performance of our previously‐designed bulk singleplex assays using a 96‐well microtiter format to establish the benchmark performance for each MethyLight assay. A standard curve was created by adding various concentrations of synthetic DNA sequences equivalent to bisulfite‐converted fully‐methylated epialleles ranging from 10 to 320 copies per reaction‐well along with TaqMan probes targeting each respective target‐locus (Figure S1, Supporting Information). To assess the specificity of the assay, a background of 10 000 synthetic copies of BST unmethylated epialleles was also added to the reaction mixture. Following PCR amplification using a standard benchtop qPCR machine, the resulting real‐time fluorescence curves showed a clear inverse correlation between copy number and PCR quantification cycle (*C*_q_). The standard curves also demonstrated R‐squared values ranging from 0.97 to 0.99, indicating excellent quantitative capability of the original singleplex MethyLight assays.

**Figure 3:**
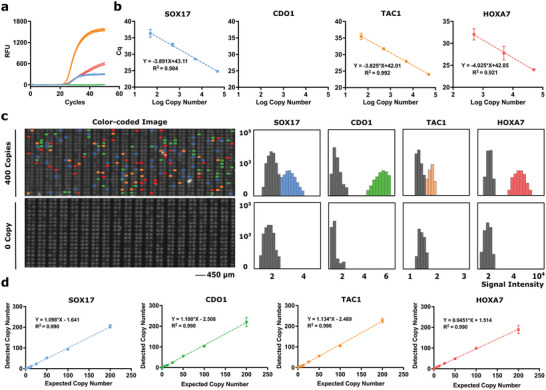
Analytical validation of bulk multiplex MethyLight and mdMSP. a) Amplification curves of *SOX17*, *CDO1*, *TAC1*, and *HOXA7* with an input of 50 000 copies. b) Standard curves of 0, 5, 50, 500, 5000, or 50 000 copies of four synthetic methylated DNA, and 10 000 unmethylated background molecules. No amplification was observed for *CDO1* in bulk. c) Representative micrographs of subsets of the merged color‐coded fluorescence images of the chip showing multiplexed detection of *SOX17* (blue), *CDO1* (green), *TAC1* (orange), and *HOXA7* (red) on chip. The population of each methylated epiallele is quantified by thresholding the signal intensity histogram. d) Detected versus expected DNA copy number. Synthetic DNA equivalents to the converted *SOX17*, *CDO1*, *TAC1*, and *HOXA7* loci ranging in copy numbers of 0, 6.25, 12.5, 25, 50, 100, or 200 are mixed with 2 00 000 unmethylated background molecules. Each experiment was done in duplicate to test the reproducibility of each assay.

Following initial assay validation, we attempted to directly combine all four singleplex MethyLight assays into a multiplex assay by combining all primers and probes together. The standard curve was then constructed with all four targets included at each concentration. Unsurprisingly, we observed that the analytic performance suffered as indicated by reduced sensitivity or even failed detection for each of the targets. Specifically, we observed decreased analytical sensitivity or limit of detection, defined as the lowest number of detectable methylated targets, in the *SOX17*, *TAC1*, and *HOXA7* assays, and the limit of quantification, defined as the lowest copy number detected in all assay replicates, was significantly higher than those of the traditional singleplex assays, particularly for *SOX17* and *TAC1* (**Figure** **3**b, **Table** **1** and Figure S2, Supporting Information). We also observed that *CDO1* failed to amplify at all concentrations tested (Figure 3 and Figure S2, Supporting Information). We attempted to specifically isolate the source of the amplification failure by retesting the standard curve of *CDO1* targets in the absence of other targets. The results of this bulk MethyLight analysis demonstrated that *CDO1* could be successfully amplified under these conditions, indicating that failed amplification in the bulk multiplex format was likely due to nonspecific interactions between *CDO1* primers and other amplicons (Figure S3, Supporting Information). We reasoned that a digital approach might help to resolve this issue by physically isolating the amplification of *CDO1* templates from competing amplicons.

**Table 1:** Analytical performance of singleplex MethyLight, multiplex MethyLight, and mdMSP

	Assay	R^2^	Limit of detection^a)^	Limit of quantification^b)^	Analytical specificity^c)^
SOX17	Singleplex (bulk)	0.805	12.5	25	0.013%
	Multiplex (bulk)	0.801	50	100	0.05%
	mdMSP (digital)	0.990	1	1 (0.56 − 1.44)^d)^	0.0005%
CDO1	Singleplex (bulk)	0.807	≤6.25	50	0.025%
	Multiplex (bulk)	N/A	N/A	N/A	N/A
	mdMSP (digital)	0.990	1	1 (0.37 − 1.63)^d)^	0.0005%
TAC1	Singleplex (bulk)	0.993	≤6.25	12.5	0.0060%
	Multiplex (bulk)	0.810	≤6.25	50	0.025%
	mdMSP (digital)	0.996	1	1 (0.52 − 1.48)^d)^	0.0005%
HOXA7	Singleplex (bulk)	0.934	≤6.25	25	0.013%
	Multiplex (bulk)	0.776	12.5	25	0.013%
	mdMSP (digital)	0.990	1	1 (0.53 − 1.47)^d)^	0.0005%

The same copy numbers of synthetic DNA were used in bulk singleplex MethyLight, bulk multiplex MethyLight and mdMSP here. Synthetic DNA equivalents to the converted *SOX17*, *CDO1*, *TAC1* and *HOXA7* loci ranging in copy numbers of 0, 6.25, 12.5, 25, 50, 100, or 200 are mixed with 2 00 000 unmethylated background molecules. The raw amplification curves and standard curves of bulk singpleplex and multiplex MethyLight are shown in Figure S2, Supporting Information.

^a)^ Limit of Detection. The lowest copy number that exhibited amplification in at least one replicate;

^b)^ Limit of Quantification. The copy number that exhibited amplification in all replicates;

^c)^ Analytical specificity is calculated as the limit of quantification of synthetic methylated DNA divided by the total copy number of synthetic unmethylated DNA;

^d)^ The mdMSP provides absolute quantification capability, therefore, the Limit of Detection and Limit of Quantification are recorded as 1 with a range considering experimental standard variations at each concentration.

To test this hypothesis, we repeated the serial dilution experiment, but in digital format using the mdMSP platform. Standard curves were generated using synthetic targets at concentrations ranging from 6.25 to 200 copies in the presence of 2 00 000 unmethylated synthetic epialleles. Following PCR on chip, target amplification was indicated by a positive fluorescence signal in a given nanowell. The endpoint fluorescence signals of all nanowells are plotted in Figure 3c, which are color‐coded into positive or negative populations and converted into a signal intensity histogram. To identify positive wells, we employed a Poisson Mixture Model to the fluorescence intensity values for each fluorophore that allowed us to obtain the means and standard deviations for the negative and positive populations. Positive wells were determined from the fluorescence intensity histogram of the negative population using a five‐sigma threshold, roughly corresponding to an allowance of one false‐positive observation for every million nanowell measurements (Figure S4, Supporting Information). Incorporating these thresholds with the mdMSP results demonstrated strong linear relationships, as well as correlations, between the detected and expected copy numbers for all targets (Figure 3d). The successful amplification of all four targets at all concentrations indicated that by physically separating amplicons into nanowells with our digital approach, the PCR competition issue we encountered in bulk multiplex MethyLight could be resolved. Linear fits of the standard curve exhibited R‐squared values ≥0.99, suggesting that the mdMSP assay provides strong absolute quantitation capability. The analytical specificity of mdMSP for detection of multiple methylated epialleles was demonstrated down to fractions as low as ≈6 copies in a background of 2 00 000 unmethylated copies, or 0.0005% analytical specificity, outperforming the corresponding traditional bulk singleplex MethyLight assays, as well as the analytical performance previously reported for the MethyLight technique itself (Table 1).^[^ ^7c^ ^]^ The observed improvement in analytical performance can likely be attributed to the increase in effective concentration of target molecules in the digital format, ultimately enabling efficient detection of nearly all target template molecules. Overall, these results highlight the advantages of a digital approach for the development of multiplex PCR assays.

### 2.4 Detection of DNA Methylation in Plasma

**Figure 4:**
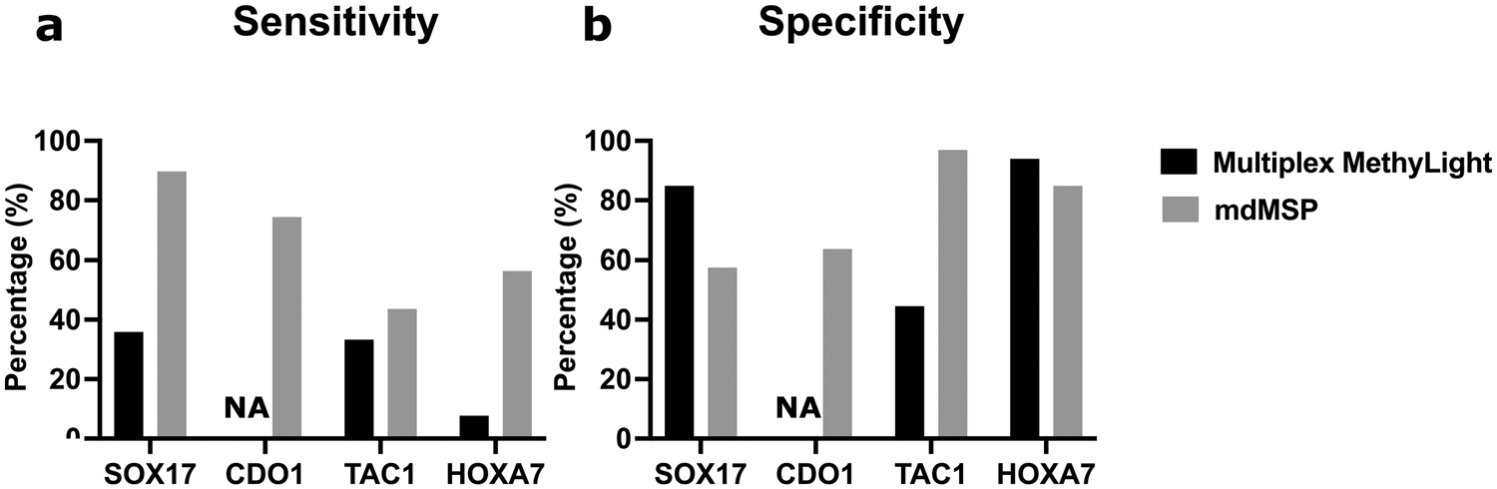
Diagnostic performance of bulk multiplex MethyLight and mdMSP. a) Clinical sensitivity of bulk multiplex MethyLight and mdMSP. The percentage indicates the fraction of positive results in patients with early‐stage NSCLC. b) Specificity of bulk multiplex MethyLight and mdMSP. The percentage indicates the fraction of negative results in patients with benign nodules. N/A indicates PCR amplification failure of *CDO1*.

We next sought to preliminarily assess the clinical potential of the lung cancer mdMSP assay for use in a liquid biopsy setting. To do this we used a study cohort comprised of plasma samples derived from liquid biopsies of 72 patients with CT‐scan identified indeterminate pulmonary nodules. 28 of these patients were later diagnosed with early‐stage (I or II) NSCLC, 11 of these patients were diagnosed with late‐stage (III or IV) NSCLC, and 33 were found to have nodules pathologically‐confirmed as benign (Table S1, Supporting Information). We also compared the clinical performance of the mdMSP assay against bulk multiplex MethyLight using bisulfite‐converted DNA isolated from 100 µL of plasma. The results of multiplex MethyLight analysis indicated that the traditional assay failed to identify a significant proportion of cancer patients, likely due to the reduced PCR efficiency or, in the case of *CDO1*, nonspecific interactions of primers with other target amplicons (**Figure** **4**a).^[^ ^15a^ ^]^ Clinical sensitivity and specificity are used to characterize the clinical performance of tests, which refer to the ability of assays to correctly identify patients with cancer or without cancer, respectively. While our previous studies indicated that the singleplex format of these assays have the ability to provide efficient detection of NSCLC with an average clinical sensitivity greater than 70% at an average specificity of 80%, the bulk multiplex MethyLight format resulted in a reduced clinical sensitivity of 30% at a specificity of 70% in this cohort.^[^ ^15b^ ^]^ In contrast, mdMSP was shown to be more sensitive than both the bulk singleplex and multiplex MethyLight formats for the detection of each of the methylated targets in the plasma of patients with cancer. While this improvement in clinical sensitivity is likely due to the superior analytical sensitivity of the mdMSP assay, this sensitivity can lead to reduced clinical specificity due to biologically noise or epigenetic drift in DNA methylation in otherwise healthy individuals. Indeed, our results indicated this was the case (Figure 4b), however, the observed reductions in clinical specificity are expected to be (and are) marker‐dependent, being nominally determined by the average prevalence of methylated copies of target cfDNA found in the blood of cancer‐free controls.

**Figure 5:**
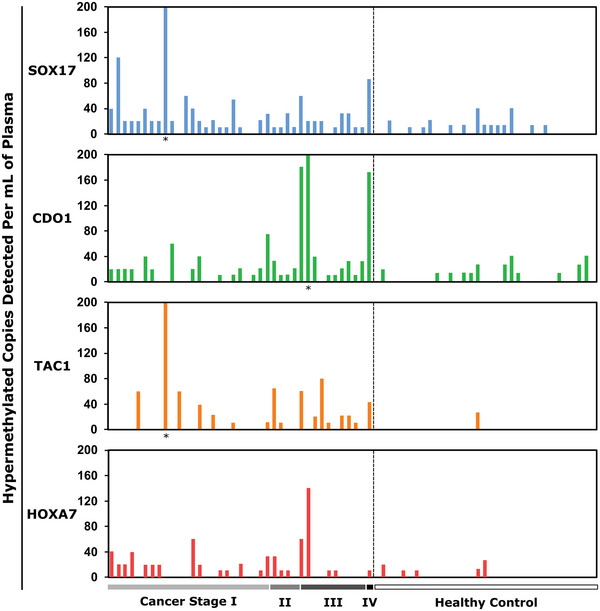
Quantitative methylation detection in plasma. mdMSP detection of *SOX17, CDO1*, *TAC1*, and *HOXA7* in plasma samples from 39 NSCLC patients and 33 cancer‐negative but CT‐positive controls. The results indicate the number of methylated copies of each target locus per mL of plasma according to sample. ^*^ >200 positive wells.

Unlike bulk assays, the mdMSP platform provides absolute‐, as opposed to semi‐, quantification of methylated epialleles, yielding the discrete number of heavily methylated epialleles per sample volume (**Figure** **5**). As expected, significantly more methylated epialleles of *SOX17*, *CDO1*, *TAC1*, and *HOXA7* were detected in cancer patients compared to the benign group. To initially compare the clinical performance between the bulk MethyLight to the mdMSP assay, we calculated the clinical sensitivity and specificity of each biomarker by employing the univariate analysis used in our prior study,^[^ ^15b^ ^]^ whereby the threshold is set to maximize the difference between the true positive rate and false negative rate (**Table** **2**). We next assessed the clinical performance of the mdMSP assay by employing multivariate analysis, which yielded a clinical sensitivity and specificity for NSCLC detection of 90% and 82%, respectively (Table 2). The detailed methodology used in multivariate analysis is described in experimental section and the full model is shown in Table S4, Supporting Information. These results compared favorably to the clinical sensitivity and specificity reported in our prior study (88% and 60%, respectively).^[^ ^15a^ ^]^

**Table 2:** Clinical performance for mdMSP‐based detection of NSCLC in plasma

Biomarker	Cancer (*n* = 39)		Control (*n* = 33)	
	*n*	Sensitivity	*n*	Specificity
SOX17^a)^	25	64%	4	88%
CDO1^a)^	21	54%	5	85%
TAC1^a)^	22	56%	0	100%
HOXA7^a)^	22	56%	5	85%
4‐marker combination^b)^	35	90%	6	82%

^a)^ Derived from univariate analysis;

^b)^ Derived from the multivariate analysis.

**Figure 6:**
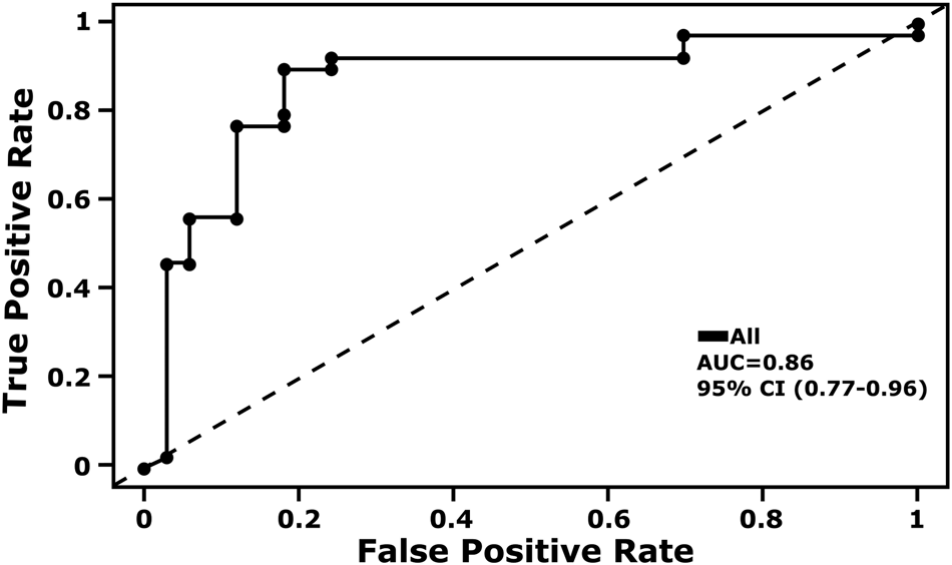
ROC performance for the detection of NSCLC from mdMSP in plasma. a) ROC curve showing the diagnostic performance for detection of NSCLC based on mdMSP analysis of *SOX17, CDO1*, *TAC1*, and *HOXA7* methylation in cfDNA from 100 µL of plasma of patients with indeterminant nodules identified by low‐dose CT screening.

Validation of the receiver operating characteristic (ROC) curve for the combination of all four biomarkers was achieved using leave‐one‐out cross‐validation with a logistic regression model and yielded an area under the curve (AUC) of 0.86 (95% CI, 0.77–0.96) (**Figure** **6**). We also found that utilizing all four biomarkers yielded better clinical performance than any individual gene or other combination of biomarkers (Table S3, Supporting Information). Overall, the performance of the 4‐marker mdMSP assay was superior to that observed for both the bulk multiplex assay (AUC 0.44, 95% CI, 0.31–0.57) (Figure S6, Supporting Information) and the previously‐published singleplex data with three best performing loci (AUC 0.77, 95% CI, 0.68–0.86). As tobacco use and chronological age have previously been reported to be important risk factors for lung cancer,^[^ ^19^ ^]^ we evaluated whether adding smoking pack years or age as predictive features could improve the performance of the logistic regression model. Results of this analysis indicated that incorporation of smoking pack years and age features did not significantly alter performance, yielding a sensitivity of 82% and 88% at a specificity of 77% and 94%, respectively, and an AUC of 0.86 (95% CI, 0.76–0.96) and 0.86 (95% CI, 0.77–0.97), respectively. Overall, our data provide strong evidence that conversion of bulk singleplex or multiplex MSP assays to a digital multiplex format can indeed provide substantial benefit in terms of analytical performance, with improved analytical sensitivity and specificity, increased linearity, and more accurate quantitation, leading to improved clinical performance for disease detection.

## 3 Discussion

Lung cancer remains the leading cause of cancer death in the US, accounting for nearly 2 36 000 lives lost every year.^[^ ^11^ ^]^ Numerous studies have demonstrated that detection at an early clinical stage has the potential to dramatically reduce mortality from the disease.^[^ ^20^ ^]^ Toward this end, it is currently recommended that persons such as heavy smokers, who are at an elevated risk of developing lung cancer, undergo periodic screening by low‐dose CT in an effort to detect potentially cancerous lesions in the lungs at early stages of disease.^[^ ^13c,e^ ^]^ While low‐dose CT has indeed demonstrated the ability to reduce lung‐cancer associated mortality rates by as much as 20%, this approach has several notable drawbacks when used as a standalone screening assay. Most notably, beyond the considerable cost, low‐dose CT has what many consider to be an unacceptably high false‐positive rate in the range of 96%–98%.^[^ ^3a,c,e,f^ ^]^ These false positive results can have detrimental effects on patient care by leading to psychological stress and expensive follow up tests such as biopsy procedures that pose risks to patients. There thus remains a considerable need for new tests with sufficiently high diagnostic performance that can be used in conjunction with low‐dose CT screening to improve overall clinical specificity and reduce the burden associated with false positive results.

Liquid biopsies have engendered substantial interest as a promising approach for the detection and monitoring of many types of cancer, including NSCLC. In our prior work, we identified a panel of four epigenetic biomarkers, namely *SOX17*, *CDO1*, *TAC1*, and *HOXA7*, which were shown to be recurrently methylated in NSCLC and can be readily detected in plasma.^[^ ^15b^ ^]^ In this study, we aimed to develop a simple multiplexed digital MSP approach that would provide parallelized detection of this panel for use in conjunction with low‐dose CT for improved screening of lung cancer in high‐risk populations. Toward this end, we developed a digital multiplex platform called mdMSP that offers improved sensitivity, analytical specificity and quantitation, even in low‐volume plasma samples. Using the mdMSP platform, we were able to successfully demonstrate multiplex analysis of all four biomarkers, achieving single‐copy sensitivities and analytical specificities as low as 0.0005% (1 in 2 00 000), which surpass the performance for the previously‐reported singleplex MethyLight assays.^[^ ^7^, ^15^ ^]^ In terms of clinical performance, the mdMSP assay demonstrated a 90% clinical sensitivity at 82% specificity, yielding a corresponding AUC of 0.86, while requiring only 100 µL of patient plasma. This compared favorably to the analogous, previously‐reported bulk singleplex assays exhibiting 93% sensitivity at 62% specificity and an overall AUC of 0.77.^[^ ^15b^ ^]^

While the clinical performance of the mdMSP assay requires further validation in a large scale study, taking the results reported here at face value would translate into a reduction in the false‐positive rate of lung cancer CT screening by 79%. In principle, these results imply that the 4‐marker multiplex assay could prevent many patients with false positives from CT‐scans from needing to go through invasive, and potentially dangerous, follow‐up procedures. The development of a liquid biopsy‐based assay emphasizes the potential clinical utility of our mdMSP platform as a means of improving patient outcomes and decreasing healthcare costs.

A primary conclusion of this study was that combining singleplex Methylight assays for the top‐performing genes provided higher diagnostic performance than any individual locus on its own. While such a multi‐target assay approach may offer improved performance, it also presents a number of technical challenges that must be overcome to improve its attractiveness for clinical applications. For example, the volume and DNA quantity of a given sample may not be sufficient to split among multiple reactions, nominally resulting in missed information or an inability to perform the assay altogether. Furthermore, assessment of each biomarker in the panel reduces throughput and can lead to run‐to‐run variability or user error that may significantly affect the reproducibility and consistency of results. On the other hand, a natural solution might be to combine the assays into a single multiplex assay, but this approach presents other technical challenges and drawbacks that can reduce overall assay performance, as demonstrated in the present study. Specifically, combining multiple singleplex assays can lead to PCR competition that leads to decreased sensitivity. Overcoming this hurdle can be tedious and challenging, often requiring several design iterations that may alter the target loci and compromise the resulting analytic and diagnostic performance. To address these issues, we employed a dPCR approach in an attempt to mitigate challenges related to multiplexed assay development, such as nonspecific interactions and PCR competition by isolating template molecules into individual nanowells. To the best of our knowledge, our mdMSP assay is the first to successfully demonstrate multiplexed dMSP‐based detection of a panel of four DNA methylation biomarkers for the early‐stage detection of NSCLC. Indeed, the superior performance demonstrated by our mdMSP approach potentially serves as a paradigm for the development for direct adaptation of other singleplex PCR assays to a multiplex [digital] format.

One notable advantage of the mdMSP platform is the use of so‐called “soft lithography” that employs PDMS (silicone) to create a microfluidic device that is easy to fabricate and low in cost compared to commercially available stationary digital PCR platforms (e.g., Qiagen QIAcuity Digital PCR System). Additionally, and in contrast to many commercially available droplet digital PCR platforms such as the QX200 Droplet Digital PCR System (Bio‐Rad), our device does not require additional or proprietary instrumentation for sample digitization and, unlike silicon‐based chips such as used in the popular QuantStudio platform (Thermo Fisher Scientific), our transparent PDMS‐based device can be imaged with standard laboratory instrumentation. Overall, the modular nature of the mdMSP platform highlights its amenability for use with commonly‐available laboratory equipment in various laboratory settings.

There are a few notable limitations in the current study as well as opportunities for future improvement. First, while a feature of our platform is the ability to use plasma sample volumes as low as 100 µL, we did not investigate the impact of input volume on diagnostic performance. This is particularly relevant given the far higher plasma volumes, often in the range of five to ten mL (or more), that are more commonly required for NGS‐based assays. The clinical sensitivity of the mdMSP assay could presumably be improved by using larger [plasma] sample volumes, but this amendment would need to be considered in light of any risks or patient compliance associated with larger blood draws. Second, the throughput of the mdMSP platform is currently limited to the analysis of only four samples per device, which may limit its utility for high patient volume settings. In the future, throughput could be tailored to such settings by increasing the volume ratio between wells and channels and by accommodating additional modules per device. With such modifications, our next‐generation microfluidic devices are expected to have 50 000 nanowells per module and at least five modules in parallel. Third, the statistical power of our study was somewhat limited by our cohort size of 72 specimens and its retrospective design may suffer from selection bias. Consequently, more extensive tests on significantly larger prospective cohorts will be required to fully validate the clinical translatability of the lung cancer mdMSP assay for use in clinical settings in the future.

In conclusion, this study demonstrated the ability of the mdMSP approach for improving the clinical performance of DNA methylation‐based diagnostics for use in conjunction with low‐dose CT screening. Our results also provide an important paradigm of the potential advantages of a digital approach for adapting promising singleplex PCR assays to a high sensitivity multiplex format for various biomedical applications. With further development, the mdMSP assay may provide an important complement to routine CT screening leading to improved clinical care and outcomes for patients at high risk of lung cancer.

## 4 Experimental Section

### Sample Population and Plasma Extraction

The study population consists of a prospective, observational cohort, initiated in 1996 within the University of Pittsburgh (“Detection of Genetic Markers of Lung Cancer Initiation and Progression,” IRB#19 060 269). All patients signed informed consent. Patients had a CT scan for suspicion of lung cancer and referred to surgery for resection. Surgical resection with curative intent and pathological analyses of suspected lung cancer lesions were completed in all patients and staged according to revised TNM guidelines classification criteria.^[^ ^21^ ^]^ Cases had pathologically confirmed NSCLC and controls were pathologically confirmed to be benign. Pack‐years of cigarette smoking were defined as the average number of packs smoked per day times the number of years smoked. Plasma samples were collected in tubes containing sodium heparin (Becton, Dickinson and Company) and then stored at −80 °C until use.

### DNA Extraction and Bisulfite Conversion

DNA extraction from plasma was performed with NeoGeneStar Circulating DNA Purification Kit (NeoGeneStar) according to the manufacturer's protocol. Briefly, 1.0 mL of plasma was digested in a solution containing Protease Buffer and 1X Proteinase K (New England Biolabs) for 30 mins at 55—60 °C. DNA was then precipitated by isopropanol, washed by a series of decantation steps, and eluted into 30 µL of Elution Buffer. Long interspersed nuclear element 1 (LINE‐1) standards were used to estimate the overall cfDNA copy numbers with 300 nm of forward primer, 5′‐ AGG GTT TTT ATG GTT TTA GGT T ‐3′, 300 nm of reverse primer, 5′‐ ATC CCT TCC TTA CAC C ‐3′, spanning 82 bp regions, and 100 nm of probe, 5′‐ ∖6FAM∖ TTG AAT TGA TTT TGT ATA A ∖MGBNFQ∖ ‐3′. Cycling conditions were 95 °C for 5 mins, and 50 cycles of (95 °C for 30 s, 50 °C for 30 s and 72 °C for 30 s). PCR was conducted using a PCR buffer containing 16.6 mm (NH_4_)_2_SO_4_, 67 mm Tris (pH 8.8), 10 mm *β*‐mercaptoethanol and magnesium chloride to yield a final magnesium concentration of 6.7 mm, 200 µm of each deoxynucleotide triphosphate (dNTP, MilliporeSigma) and 0.04 U µL^−1^ of Platinum *Taq* polymerase (Thermo Fisher Scientific). Final reaction volumes for LINE‐1 quantification assays were 25 µL. The resulting DNA was bisulfite treated using the EZ DNA Methylation‐Lightning Kit (ZYMO RESEARCH) according to the manufacturer's instructions and eluted into a final volume of 30 µL. The yields of post‐treated cfDNA were quantified by LINE‐1 quantification assay as described above. All synthetic control DNA was purchased from Integrated DNA Technologies (IDT) and used at concentrations suggested by manufacturer.

### Singleplex/Multiplex MethyLight

Primers and hydrolysis probes were designed with Primer3 (version 4.1.0).^[^ ^22^ ^]^ Sequences of primers and probes for MethyLight analysis are listed in Table S1, Supporting Information. Multiplex quantitative real‐time Methylation Specific PCR was performed on a CFX96 Touch Real‐time PCR Detection System (Bio‐Rad) and analyzed with the CFX Manager (version 3.1). Each reaction has a final volume of 25 µL consisting of PCR buffer, 200 nm of each forward primer, 200 nm of each reverse primer, 100 nm of each probe, 200 µm of each dNTP, 0.04 U µL^−1^ of Platinum *Taq* polymerase and 2.5 µL of bisulfite converted DNA. Cycling conditions were 95 °C for 5 mins, and 50 cycles of (95 °C for 30 s, 65 °C for 30 s and 72 °C for 30 s). PCR amplification was performed on 96 well‐plates (Bio‐Rad) in triplicates. As described previously,^[^ ^15b^ ^]^ each methylation detection replicate was comparing with the mean cycle threshold (Ct) of reference gene quantification to calculate the 2^−ΔCt^. With the extreme low level of DNA methylation in plasma‐derived cfDNA, replicates with undetectable methylation were expected. To reduce the measurement bias, a Ct of 100 was used for replicates with no detected methylation, creating a close‐to‐zero value for 2^−ΔCt^. The mean 2^−ΔCt^ is calculated by,

(1) $$\bar{2^{-\Delta \mathrm{Ct}}}=\frac{\left(2^{-\Delta \mathrm{Ct}\;\mathrm{replicate}1}+2^{-\Delta \mathrm{Ct}\;\mathrm{replicate}2}+2^{-\Delta \mathrm{Ct}\;\mathrm{replicate}3}\right)}{3}$$

### Chip Fabrication

Microfluidic chips were fabricated using the previously described chip fabrication method.^[^ ^18a^ ^]^ Briefly, a clean 4 inch silicon wafer (Polishing Corporation of America) was dehydrated at 200 °C and oxygen plasma‐treated (Technics PE‐IIA) at 80 W for 1 min. SU‐8 3050 photoresist was spun at 1800 rpm for 1 min and soft‐baked at 95 °C for 30 mins. The wafer was exposed using a mask aligner at 175 J cm^−2^ allowing pattern transfer from a photomask, developed in SU‐8 developer, and baked at 200 °C for 2 h.

Each microfluidic chip was fabricated from this mold using soft lithography and ultrathin 80 µm layering technique.^[^ ^18a^ ^]^ The wafer was treated with chlorotrimethylsilane (Sigma‐Aldrich) in a vacuum chamber for 2 mins to increase the hydrophobicity of silicon wafer surface. A 15:1 mixture of PDMS (Ellsworth) was spun on wafer surface at 500 rpm and a 6:1 mixture of PDMS was spun on a blank wafer as a sacrificial layer. Both were baked for 6 mins at 80 °C. The sacrificial layer was peeled off and overlaid on pattern surface and then baked for 6 mins at 80 °C. A large microscope glass slide (75 mm × 50 mm × 1 mm thick; Ted Pella) was cleaned and air‐dried. A 15:1 mixture of PDMS was spun on the glass slide at 2100 rpm and then baked at 80 °C for 6 min. The patterned‐sacrificial‐jointed layer and the glass slide were oxygen plasma‐treated at 40–45 W for 45 s and then bonded. After bonding, the sacrificial layer was removed. Subsequently, a thin cover glass slide and tubing adapter layer were oxygen‐plasma‐bonded to the top surface. The fabricated devices were baked at 80 °C overnight, sealed with a piece of thin adhesive tape (Scotch) and desiccated for at least 3 h.

### Sample Loading and Digitization

Each multiplex digital PCR master mix has a 20 µL of PCR mixture consisting of PCR buffer, 200 nm of each forward primer, 200 nm of each reverse primer, 100 nm of each probe, 200 µm of each dNTP, 0.04 U µL^−1^ of Platinum Taq polymerase, 1 mg mL^−1^ of bovine serum albumin (BSA) (New England BioLabs), 0.01% Tween 20 (Sigma‐Aldrich) and 5 µL of bisulfite converted DNA sample. Sample loading and digitization were conducted using the previously described approach.^[^ ^18a^ ^]^ Briefly, a 1 mL syringe (BD Syringe) storing master mix was used to puncture the sealed inlet and the negative pressure difference presenting in a sealed microfluidic chip allowed sample loading. The partitioning fluid, consisting of 5 g of silicon oil and a 1 g of 10:1 mixture of PDMS, was drawn into a microcentrifuge tubing (Cole‐Parmer) and then pressurized into the channels of a microfluidic chip. When partitioning fluid flowed through channels the sample was digitized throughout the 10 040 wells of each module. The volume of PCR master mix remained in nanowells accounts for ≈50% of the total master mix prepared. Sample loading efficiency would be significantly improved by increasing the volume ratio between microchambers and channels in the next generation of device.

### Digital PCR

To conduct digital PCR, the chip was placed on a flatbed thermal cycler (Bulldog Bio) with partitioning fluid pressurized at one end at ≈12 psi and sealed at the other end. FC 40 oil was used to fix the device on the surface of the thermal cycler. The cycling conditions of digital PCR were 95 °C for 5 mins, and 50 cycles of (95 °C for 30 s, 63 °C for 30 s, and 72 °C for 30 s). Because of the addition of PDMS components, the partitioning oil solidified during PCR cycles, providing permanent barriers to prevent leakage of sample from nanowells to channels even without pressurization.

### Image Acquisition and Processing

After PCR was completed, the microfluidic chip was disconnected from the pressure regulator, removed from the thermal cycler, and then scanned with an Amersham Typhoon 5 Biomolecular Imager (GE Healthcare). Amersham Typhoon 5, equipped with LED light sources and accompanying filters for wavelength ranging from 390 to 840 nm, meets with the image acquisition requirements. Average fluorescence intensity from the nanowell was extracted using a custom‐developed program written in MATLAB as previously described.^[^ ^23^ ^]^ The positive wells were identified by fitting a Poisson Mixture Model to the fluorescence intensity histogram for each fluorophore to obtain the means and standard deviations for the negative and positive populations. Positive wells were determined by using a five‐sigma threshold of the negative population.

### Univariate and Multivariate Analysis

Logistic regression was used to build a model for predicting cancer status based on the DNA methylation levels of individual one marker (univariate) or any marker panel combinations (multivariate). The full model of multivariate analysis is shown in Table S4, Supporting Information. To minimize the effects of overfitting, performance was calculated with a leave‐one‐out cross‐validation design. The input entering the ROC computation was the predicted probability of each observation validation sample to fall into either of case or control class assigned to the sample by the algorithm. Each of these probabilities would be considered as different classification thresholds to identify the number of true and false positives and negatives. These numbers of true and false positives and negatives were converted into true positive rate and false positive rate as shown in the ROC curves. Performance was assessed by an AUC and reported with bootstrap 95% confidence intervals (95% CI). All tests were performed using the R statistical software suite (available at http://www.r‐project.org) with both standard packages and custom code.

## Conflict of Interest

The authors declare no conflict of interest.

## Supporting information

Supporting Information

## Data Availability

The data that support the findings of this study are available from the corresponding author upon reasonable request.
