# Supplementary material for: Multiplex Digital Methylation‐Specific PCR for Noninvasive Screening of Lung Cancer
Source: Adv Sci (Weinh). 2023 Apr 11;10(16):2206518. doi: 10.1002/advs.202206518 (PMC10238189; doi:10.1002/advs.202206518)
Supplement: Supplementary file 1 — Supporting Information [file ADVS-10-2206518-s001.pdf]

Supporting Information

Multiplex digital methylation-specific PCR for noninvasive detection of lung cancer

Yang Zhao+, Christine M. O'Keefe+, Kuangwen Hsieh, Leslie Cope, Sonali C. Joyce, Thomas R. Pisanic, James G. Herman, Tza-Huei Wang**

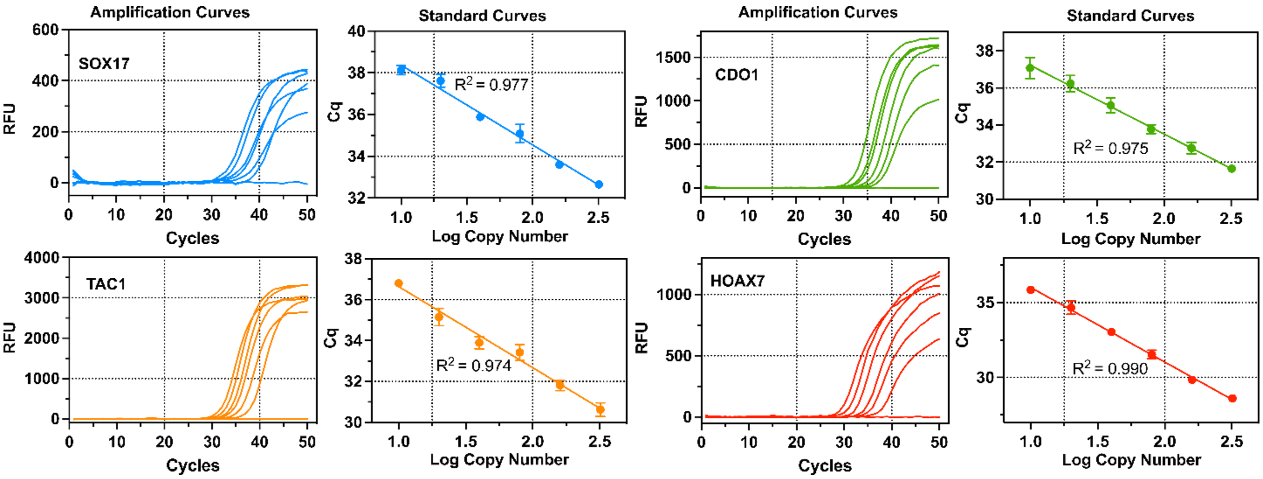

Figure S1. Singleplex MethyLight

Left: Amplification curves of *SOX17*, *CDO1*, *TAC1*, and *HOXA7*, respectively. Right: Standard curves of *SOX17*, *TAC1*, and *HOXA7*, respectively. Each reaction well contained 0, 10, 20, 40, 80, 160 or 320 copies of synthetic DNA equivalents to the BST loci mixed with 200,000 unmethylated background molecules.

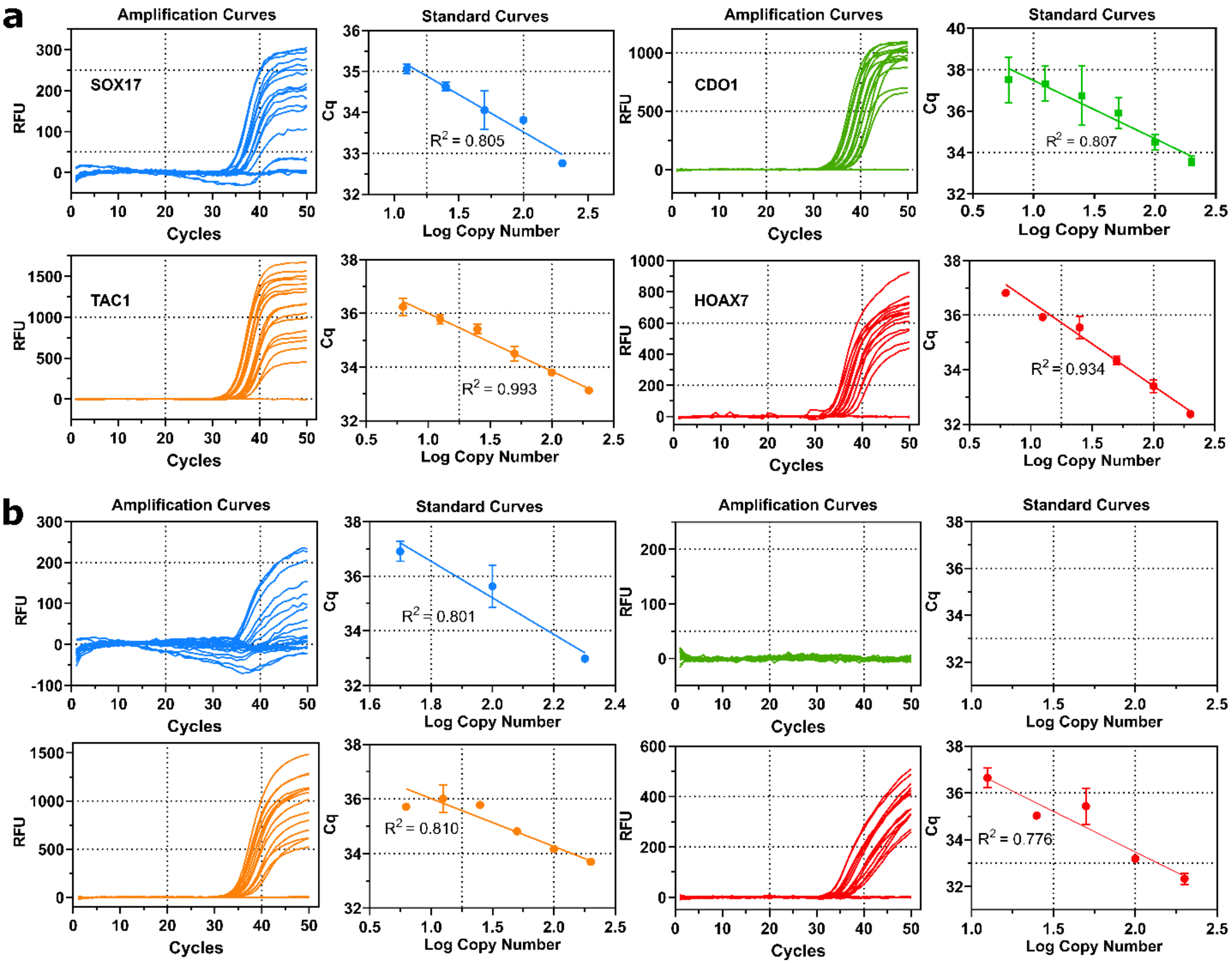

Figure S2. Bulk MethyLight conducted with the same setup as mdMSP

(a) Bulk singleplex MethyLight. (b) Bulk multiplex MethyLight. Left: Amplification curves of *SOX17*, *CDO1*, *TAC1*, and *HOXA7*, respectively. Right: Standard curves of *SOX17*, *TAC1*, and *HOXA7*, respectively. Each well contains 0, 6.25, 12.5, 25, 50, 100, 200 copies of synthetic DNA equivalents to the BST loci mixed with 200,000 unmethylated background molecules.

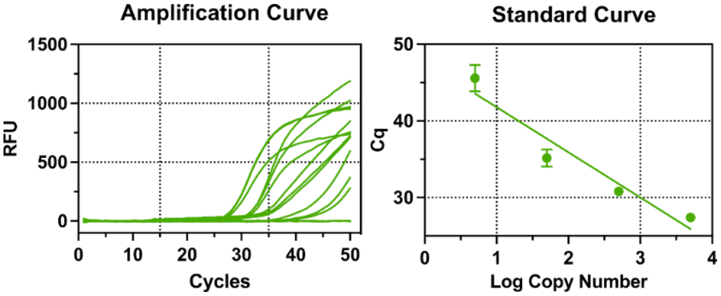

Figure S3. *CDO1*-only detection in bulk multiplex assay

Left: Amplification curve of *CDO1*. Right: Standard curve of *CDO1*. The qPCR results of *CDO1* at 0, 5, 500, 5000 copies of synthetic methylated DNA oligonucleotide. The successful amplification indicated that the failure in multiplex MethyLight (Figure S2) is likely due to nonspecific interactions between *CDO1* primers/probe and other amplicons but not primer-dimer formation.

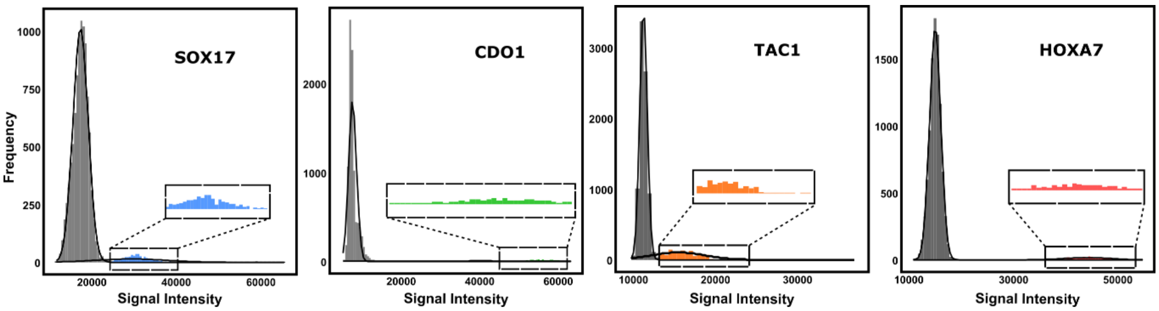

Figure S4. Poisson mixture model for setting threshold

Poisson Mixture Model was fitted on intensity histogram of each target to obtain the mean and standard deviation for the negative population. A five-sigma result from the negative population (negatives are labeled in grey) was used as a threshold to determine the number of positive wells.

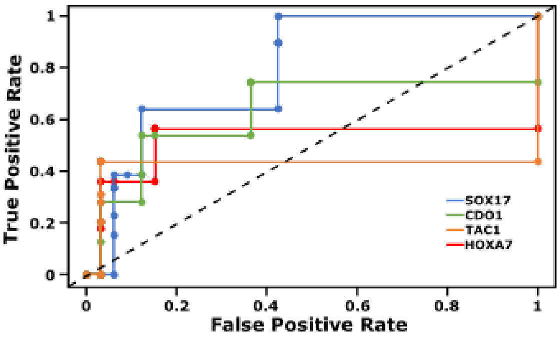

Figure S5. ROC curves of each target for mdMSP

ROC curves of the detection of NSCLC in plasma based on mdMSP analysis of *SOX17*, *CDO1*, *TAC1* and *HOXA7*, respectively.

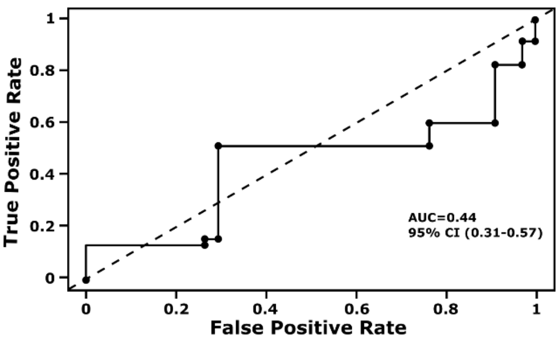

Figure S6.

ROC performance for the detection of NSCLC from bulk multiplex MethyLight in plasma

ROC curve showing the diagnostic performance for detection of NSCLC from bulk multiplex MethyLight analysis of *SOX17*, *CDO1*, *TAC1*, and *HOXA7* methylation in cfDNA from 100 µL of plasma of patients with indeterminant nodules identified by low-dose CT screening.

Table S1 Patient characteristics

Patient Characteristics	Cancer (n = 39)	Control (n = 33)	*p* Value
Age at surgery (years; IQR)	68 (42 – 78)	62.5 (23 – 81)	0.001*
Gender			
Male (%)	18 (46%)	19 (58%)	
Female (%)	21 (54%)	13 (39%)	0.267**
Not Stated (%)	0	1 (3%)	
Smoking status			
Current (%)	18 (46%)	10 (30%)	
Former (%)	20 (51%)	7 (21%)	<0.0001**
Never (%)	1 (3%)	15 (45%)	
Not stated (%)		1 (3%)	
Stage			
I (%)	24 (62%)		
II (%)	4 (10%)	N/A	N/A
III (%)	10 (26%)		
IV (%)	1 (2%)		
Histology			
Adenocarcinoma (%)	22 (56%)		
Squamous cell (%)	15 (38%)	N/A	N/A
Large cell (%)	1 (3%)		
Not stated (%)	1 (3%)		
Pack-year (IQR)†	40 (4 – 180)	30 (4 – 62)	<0.0001*

Abbreviations: IQR, interquartile range; NA, nonapplicable. *Wilcoxon test was used. **Chi-squared test was applied. †Smoking pack-year was calculated in ever smokers only.

The *t*-test was used to determine whether smoking affects methylation level of each biomarker within the control group. *P*-values ranging from 0.333 to 0.657 indicates the difference is not significant.

Table S2. Primers and probes of MethyLight/mdMSP.

Gene	Forward 5' – 3'	Reverse 5' – 3'	Probe
SOX17	TTGGATTGGGACG TGGGATTCG	GAAAACGAACCG ATCCCGCG	/56-FAM/GTGGGTTTA/ZEN/ACGA CGCGGGATCGGTTCG/3IABkFQ/
CDO1	CGTTTTTTTTCGTT TTATTTTCGTCG	CCTCCGACCCTTT TTATCTACG	/5HEX/TGTGGTTCG/ZEN/CGACGTTG GGACGT/3IABkFQ/
TAC1	TCGGGTTATTTCG TTTCGTATTTGTTC	CACTATCCCTCG CCGCAACG	/5TexRd-XN/AGGTGGTCGCGTTG GGGGCGTCGT/3IAbRQSp/
HOXA7	TTTGGAGGTTTCG CGGGAG	CGAACCGCCTAA CGTCCG	/5Cy5/TTTAGTTGG/TAO/CGGTTAATT TTCGTATTGGGGTTTGCG/3IAbRQSp/

Table S3. Clinical performance of all possible biomarker combinations for mdMSP-based detection of NSCLC in plasma.

Gene(s)	Sensitivity	Specificity	AUC	95% CI
One Gene				
SOX17	64%	88%	0.742	0.622-0.862
CDO1	54%	85%	0.626	0.488-0.763
TAC1	56%	100%	0.577	0.422-0.732
HOXA7	56%	85%	0.521	0.370-0.672
Two Genes				
CDO1 + TAC1	77%	85%	0.772	0.652-0.892
CDO1 + HOXA7	72%	76%	0.688	0.557-0.820
CDO1 + SOX17	85%	73%	0.789	0.679-0.898
TAC1 + HOXA7	82%	85%	0.763	0.634-0.891
TAC1 + SOX17	74%	88%	0.815	0.710-0.920
HOXA7 + SOX17	79%	82%	0.790	0.680-0.901
Three Genes				
CDO1 + TAC1 + HOXA7	82%	88%	0.839	0.733-0.944
CDO1 + TAC1 + SOX17	79%	88%	0.848	0.752-0.945
CDO1 + HOXA7 + SOX17	90%	67%	0.806	0.700-0.911
TAC1 + HOXA7 + SOX17	85%	88%	0.848	0.748-0.949

Four Genes				
CDO1 + TAC1 + HOXA7 + SOX17	90%	82%	0.864	0.771-0.958

Table S4. Logistic regression model on all samples.

	Coefficient Estimate	Standard Error	*z* Value	*p* Value
Intercept	-1.997	0.563	-3.547	<0.001
SOX17	0.049	0.025	1.936	0.053
CDO1	0.041	0.025	1.634	0.102
TAC1	0.096	0.050	1.899	0.058
HOXA7	0.087	0.041	2.151	0.031
